# Mechanofluorochromism and self-recovery of alkylsilylpyrene-1-carboxamides†

**DOI:** 10.1039/d3tc03968d

**Published:** 2024-01-19

**Authors:** Yuichi Hirai, Anna Wrona-Piotrowicz, Janusz Zakrzewski, Magdalena Ciechańska, Takahito Ohmura, Takashi Takeda, Takayuki Nakanishi, Rémi Métivier, Clémence Allain

**Affiliations:** a National Institute for Materials Science (NIMS) 1-1 Namiki Tsukuba 3050044 Japan hirai.yuichi@nims.go.jp; b Department of Organic Chemistry, Faculty of Chemistry, University of Łódź Tamka 12 91-403 Łódź Poland; c Université Paris-Saclay, ENS Paris-Saclay, CNRS, PPSM 4 Avenue des Sciences 91190 Gif-sur-Yvette France remi.metivier@ens-paris-saclay.fr clemence.allain@ens-paris-saclay.fr; d National Institute for Materials Science (NIMS) 1-2-1 Sengen Tsukuba 3050047 Japan

## Abstract

A family of (alkylsilyl)pyrene-1-carboxamides exhibits similar mechanofluorochromic responses upon grinding. However, their spontaneous fluorescence recovery processes are distinct despite their similarity in chemical structures and crystal packings. Fluorescence spectroscopy, crystallography, and nanomechanical tests revealed that the chromic direction is dominated by the packing motif, while the fluorescence recovery is driven by the intermolecular interactions and the reversibility of deformation.

## Abstract 2

The stimuli-responsive properties of mechanofluorochromic pyrene derivatives, including spontaneous solid-state recovery processes, are elucidated through crystallography, spectroscopy, and nanomechanics.

## Graphical abstract

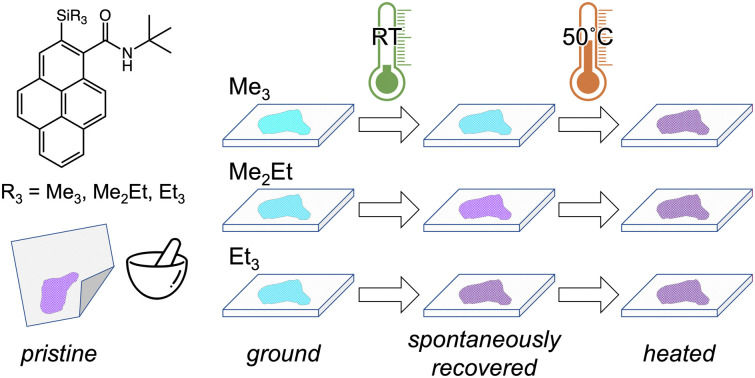

## Introduction

Mechanofluorochromism (MFC), the phenomenon where organic and coordination compounds exhibit reversible changes in their emission properties under mechanical stimuli, has emerged as a captivating area of research in materials science, chemistry, and photonics.^1–3^ Thus, it has garnered considerable attention owing to its potential applications in sensors to visualise the location/magnitude of the stress and information encryption systems for anticounterfeiting technologies.^4,5^

The ability to modulate the emission properties of compounds through mechanical deformation can be applied to the design of smart materials with tuneable and responsive luminescent behaviour. When subjected to external mechanical stimuli, such as compression, shear, grinding, or stretching, MFC materials experience changes in discrete molecular conformations, intermolecular interactions, and corresponding electronic and vibrational properties, which in turn manifest as shifts in their emission wavelength, intensity, and decay dynamics.^6,7^ One of the primary drivers of MFC is the reversible breaking and formation of non-covalent interactions, such as π–π stacking, hydrogen bonding, or metallophilic interactions, which play a crucial role in the molecular packing and electronic communication within the material.^8–10^ In addition to the disruption or re-establishment of these interactions under mechanical stress, the presence of twisted intramolecular charge transfer states and aggregation-induced emission (AIE) units can also contribute to the MFC response, thereby enhancing the emission efficiency and spectral shifts upon deformation.^11–13^ Thus, understanding the fundamental principles governing MFC and elucidating structure–property relationships are crucial for designing and optimising materials with tailored MFC behaviour.

To investigate these relationships, we focused on a series of pyrene-derived compounds by introducing different substituents to realise diverse steric and electronic structures.^14–16^ These structural modifications induce changes in the intermolecular interactions and molecular flexibility/rigidity, which influenced the mechanical response and consequent luminescent properties. While relevant research primarily focuses on large-wavelength shifts and multi-functionality,^17–20^ there have been rare comprehensive demonstrations of the intricate interplay between molecular structures and their dynamic mechanical/photophysical responses in a bulk solid state, including spontaneous recovery processes.^21^

**Fig. 1:**
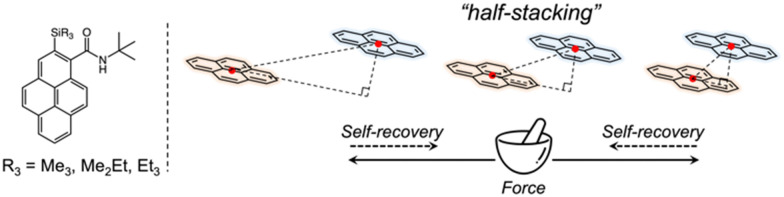
Chemical structures of pyrene-derived carboxamides with alkylsilyl groups, and a schematic of the stacking model.

In this study, we focused on a family of pyrene-derived carboxamides with alkylsilyl groups^22^ because of their reported monomer-like purple fluorescence in the solid state and the characteristic “half-stacking” arrangement, which allows realising MFC by increasing or decreasing the effective π-orbitals overlaps (Fig. 1). We investigated the fluorescence responsivity from a broad perspective using crystallography, spectroscopy, and nanomechanics to guide the design and development of novel materials with enhanced MFC properties.

## Results and discussions

**Fig. 2:**
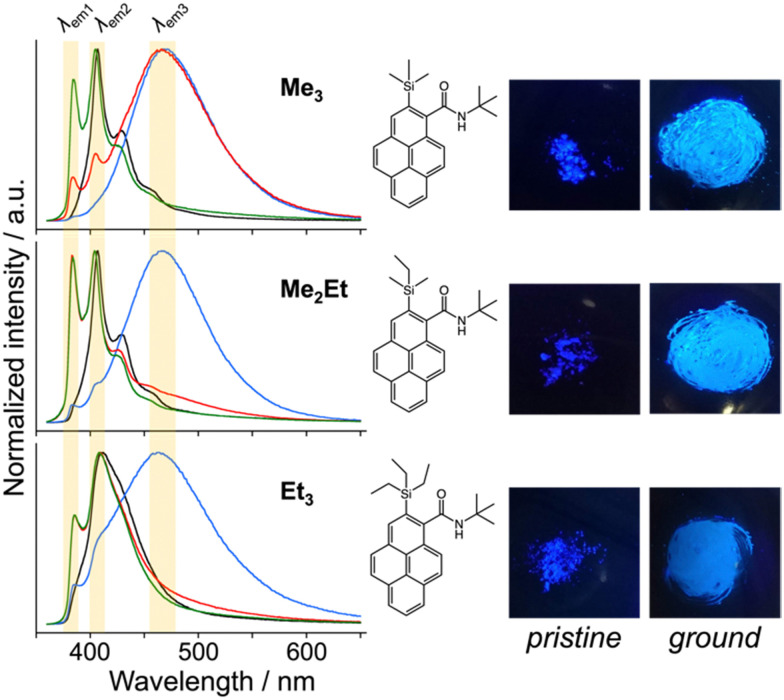
Normalised emission spectra of Me_3_, Me_2_Et, and Et_3_ at room temperature (RT) (black: pristine, blue: ground, red: 30 min at RT after grinding, green: 30 min at RT and 30 min heating at 50 °C after grinding, *λ*_ex_ = 340 nm, in the solid state), and chemical structures and photographs under UV irradiation (left: pristine, right: ground, *λ*_ex_ = 365 nm). Emission bands at approximately 388, 405–410 and 460 nm are highlighted by yellow shadows.

*N-tert*-butyl-2-(alkylsilyl)pyrene-1-carboxamides (alkyl = (CH_3_)_3_, (CH_3_)_2_(C_2_H_5_) and (C_2_H_5_)_3_) were synthesised following a reported method^22^ and denoted as Me_3_, Me_2_Et, and Et_3_, respectively. The pristine powders exhibited purple fluorescence, and the recorded profiles (Fig. 2, black lines) were the same as those reported previously; *λ*_em2_ was in the range of 405–410 nm, and a slightly broader spectrum was observed in the case of Et_3_ where the vibrational structure is attenuated. As mentioned already, the “half-stacking” arrangement of the π-orbitals of Me_3_ contributed to the monomeric emission bands, even in the solid state. Thus, the same stacking is expected for Me_2_Et, and the less-dominant monomeric emission bands in Et_3_ indicates the larger overlapping of π-orbitals. Upon mechanical grinding using a pestle and a mortar, a purple-to-blue bathochromic MFC was observed for all compounds, which indicates the formation of aggregated conformations with new intermolecular interactions. MFC spectroscopy was performed following the reported procedures to determine the mechanical responsivity including spontaneous self-recovery processes in the solid state (Fig. S1, ESI†).^16^ An increased broad emission band (*λ*_em3_ ∼ 460 nm) and accompanying near-ultraviolet (UV) shoulder bands (*λ*_em1_ ∼ 388 nm) with different degrees of contribution were observed (Fig. 2, blue lines). The relative intensity of the near-UV bands was minimal immediately after grinding and increased over time, particularly for Me_2_Et and Et_3_ (Fig. S2, ESI†). The shoulder bands should stem from the residues of the pristine and/or the ground forms after undergoing fast spontaneous molecular rearrangements under ambient conditions. In this study, the self-recovery rates were compared qualitatively by the spectral intensity ratios of the ground forms at *λ*_em2_ and *λ*_em3_, which resulted in the order of Me_3_ < Me_2_Et ≪ Et_3_. The spectral feature was almost preserved for Me_3_ after 30 min at room temperature, whereas the fluorescence spectra of Me_2_Et and Et_3_ showed a remarkably diminished emission band at 460 nm and concomitant increase in the UV emission for the same duration (Fig. 2, red lines). All compounds exhibited pristine-like spectra with an additional peak at approximately 388 nm after heating (Fig. 2, green lines), which are denoted as “recovered” in the remainder of the text. This indicates the mobility of the compounds in the solid state for reverting back to the pristine form and beyond without the aid of organic vapours or solvents. Differential scanning calorimetry (DSC) of pristine powders also indicated the lack of phase transitions under the condition of the MFC recovery processes (Fig. S3, ESI†).

**Fig. 3:**
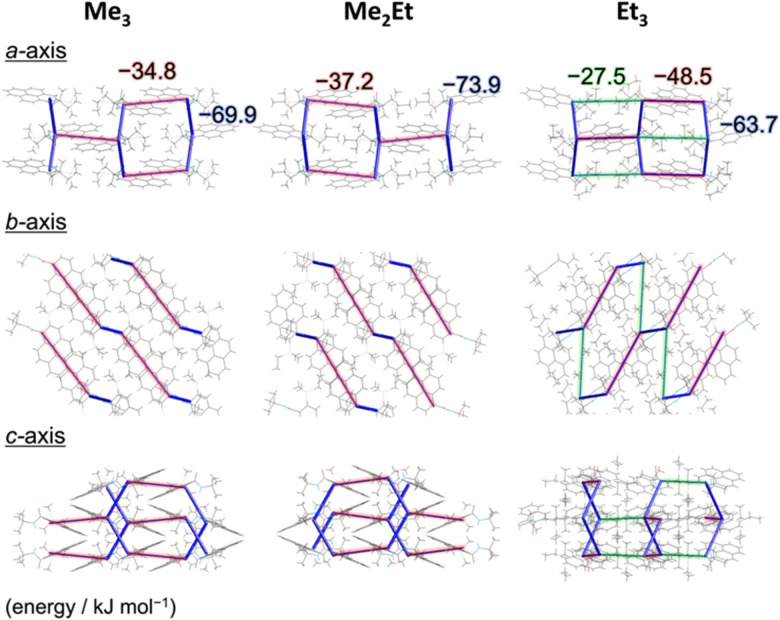
CE-B3LYP energy frameworks of Me_3_, Me_2_Et, and Et_3_ from different perspectives representing the net interaction energy *E*_InterMol_ (cut-off: 25.0 kJ mol^−1^) at 123 K. The blue cylinders connect the centres of mass of adjacent molecules, and the diameter of cylinders are proportional to the magnitutdes of *E*_InterMol_. Opaque red and green shadows highlight the interactions between parallel half-stacked pairs and non-parallel head-to-head pairs without NH⋯O=C hydrogen bonding, respectively.

To correlate the molecular arrangement and MFC response, the crystal structures of all compounds were determined using single-crystal X-ray diffraction (XRD) measurements and those of Me_3_ were re-determined. The space group was determined to be *P*2_1_/*c* with half-stacking features, and the coincidence of simulated and experimental powder XRD (PXRD) patterns confirmed the phase purity of the pristine powders (Fig. S4, ESI†). The packing manner was categorised as a herringbone motif, which has atomic contact ratios %C⋯H/%C⋯C > 4.5, *via* the Hirshfeld surface analysis^23–25^ for all molecules (Fig. S5, ESI†). This corroborates the purple-to-blue bathochromic MFC behaviours of Me_3_, Me_2_Et, and Et_3_, where the monomer-like conformation shifts to a dimer-/aggregated-like conformation under mechanical stimuli.^16^ However, the similar values of atomic contacts between adjacent molecular surfaces and packing motifs still do not explain the distinct kinetic gap in the self-recovery processes that spontaneously occur after unloading. An energy framework analysis was also performed using CrystalExplorer to quantitatively visualise the topology of the intermolecular interactions in these crystals.^26–29^ The three-dimensional (3D) topologies illustrated the strongest interaction energy (*E*_InterMol_ of approximately −70 kJ mol^−1^) between head-to-head pairs that involve NH⋯O=C hydrogen bonding in all cases, which dominates the crystal packing manner (Fig. 3). In addition to the 30–39% larger *E*_InterMol_ between half-stacked pairs compared with others, Et_3_ formed another interaction (−27.5 kJ mol^−1^) between its head-to-head pairs without NH⋯O=C hydrogen bonding. Therefore, relatively stronger and multidimensional interactions between molecules could retain fewer monomeric fluorescence features of Et_3_ and drive the faster reconstruction of molecular packing in the ground form.

**Fig. 4:**
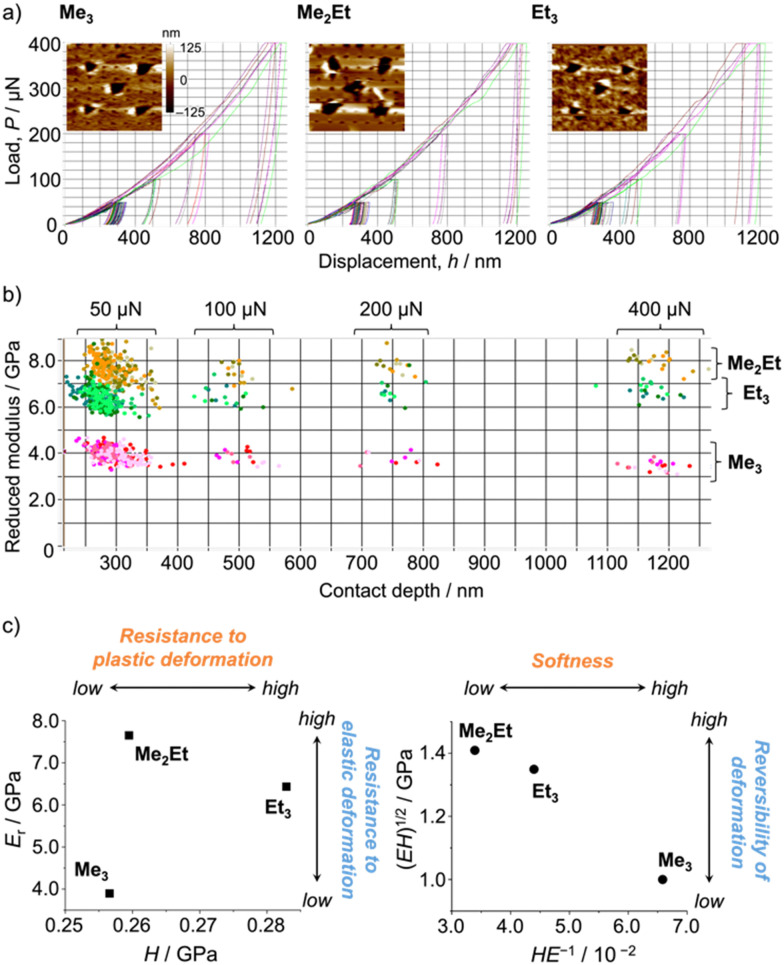
(a) Selected load–displacement curves and *in situ* scanning probe microscopy two-dimensional images after indentations (20 μm × 20 μm). (b) Distribution map of Young's modulus and the contact depth (>300 indentations for each molecule). (c) Young's modulus *versus* the hardness plot (left) and the geometric mean *versus* the elasticity index plot (right).

Following those crystallographic characteristics, the mechanical properties of the crystals were also investigated using nanoindentation experiments (see Fig. S6 in ESI† for testing procedures). The load–displacement (*p*–*h*) curves exhibited similar responses without sudden bursts (so-called “pop-ins”),^30^ which is common in brittle molecular crystals (Fig. 4a). The reduced Young's modulus (*E*_r_) and hardness (*H*) were estimated from more than 300 data sets for each molecule using the Oliver–Phar method (Fig. 4b and c, left).^31^ The estimated values indicated that Me_3_ was the softest among the studied compounds, as it exhibited relatively low *E*_r_ and *H* values (3.9 and 0.26 GPa, respectively). The slow recovery nature of softer compounds was also supported by the pioneering study on MFC using polymorphic difluoroboron avobenzone crystals with distinct plasticity.^21^ In this study, additionally, the elasticity index (*H*/*E*) and geometric mean ((*EH*)^1/2^) were introduced to reflect the type of deformation and include both stiffness and hardness (Fig. 4c, right).^32,33^ Thus, the indentation hardness of Me_3_ was dominated by a more irreversible (plastic) character than the reversible (elastic) ones present in Me_2_Et and Et_3_. Although no general mathematical relationships yet exists between stiffness and density, highly dense materials are more prone to show higher *E*_r_ values.^34^ In this case, the higher void volume of Me_3_ (*V*_void_ = 285, 266, and 267 Å^3^ for Me_3_, Me_2_Et, and Et_3_, respectively) possibly resulted in softer and more irreversible characteristics than in the other compounds because Me_3_ and Me_2_Et show similar *E*_InterMol_ distributions and all compounds have similar crystal densities. Therefore, microscopic mechanical parameters may govern some of the self-recovery trends after unloading, which explains the relatively slow fluorescence recovery in Me_3_.

**Fig. 5:**
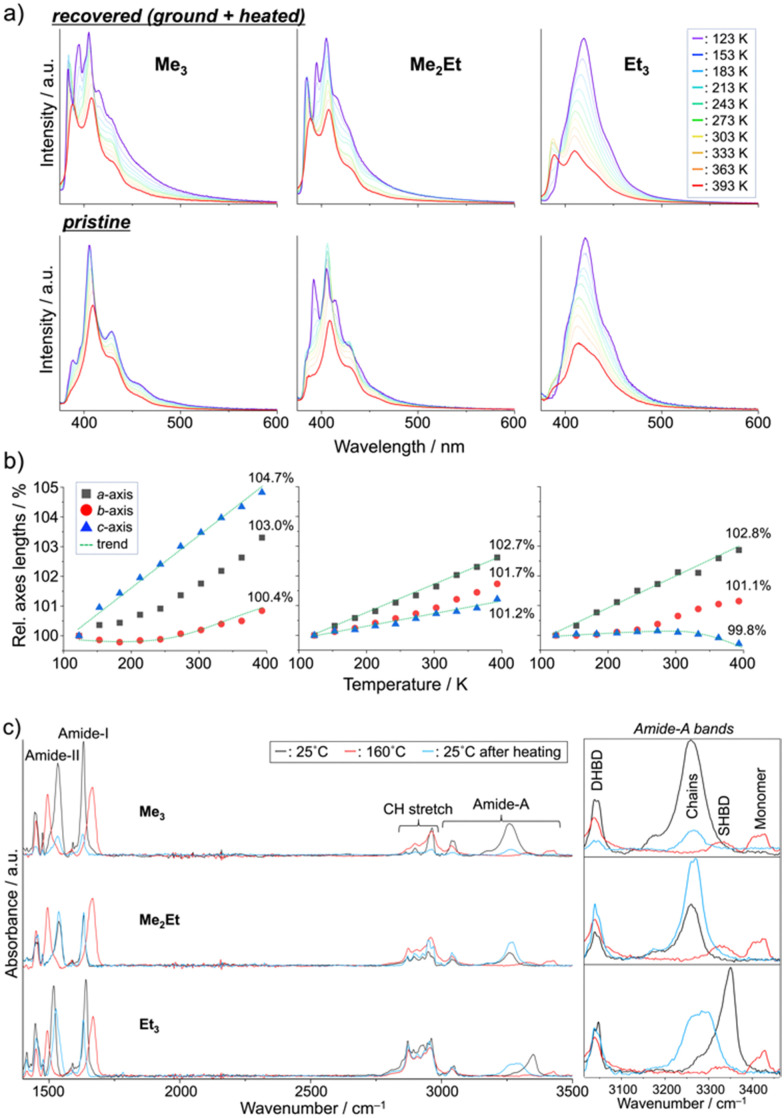
Temperature-dependent (a) fluorescence spectra (top: recovered, bottom: pristine), (b) relative unit-cell axes lengths (values at 393 K are indicated), and (c) ATR-FTIR spectra (DHBD: doubly hydrogen-bonded dimers, SHBD: single hydrogen-bonded dimers) of Me_3_, Me_2_Et, and Et_3_.

Finally, we performed *in situ* temperature-variable spectroscopy on the recovered solids and pristine powders and crystallography on the single crystals in the range of 123–393 K to consider the solid-state mobility of the molecules in terms of fluorescence signals and absolute molecular configurations. The temperature-dependent emission spectra of the recovered samples revealed nearly identical profiles and thermal responses for Me_3_ and Me_2_Et (Fig. 5a, top). In addition to the small overlap of effective π-orbitals in the pristine forms, mechanical grinding further dislocated the half-stacked pairs, thereby resulting in the typically structured fluorescence of monomeric pyrenes at low temperatures. By contrast, the recovered form of Et_3_ showed ratiometric spectral changes between the VIS (*λ*_em_ ∼ 410–419 nm) and UV (*λ*_em_ ∼ 388 nm) bands. The intensity ratios (*I*_VIS_/*I*_UV_) decreased by 15-folds upon heating, accompanying a hypsochromic shift of 9 nm of the VIS peak (Fig. S7, ESI†). Pristine Et_3_ also showed a similar response with a smaller contribution of the UV band (Fig. 5a bottom), which indicated the existence of effective stacking in both the pristine and recovered forms. Such a temperature-dependent fluorescence response based on the interchromophoric interactions in a bulk solid material was also reported in a pyrene-based metal–organic framework.^35^

Single-crystal XRD (SCXRD) experiments at different temperatures demonstrated the evolution of the unit-cell parameters upon heating (Fig. 5b and Tables S1–S3, ESI†). The contraction/expansion of crystals is generally observed under a controlled pressure using a diamond anvil cell (DAC), and the magnitude of cell expansion in this study (3–5% in the Me_3_*c*-axis, Me_2_Et*a*-axis, and Et_3_*a*-axis at 393 K relative to 123 K) is in the same order as that reported for pyrene-1-carbaldehyde (PA) at several GPa in DAC (relative to the atmospheric pressure).^36^ As previously mentioned for the emission of PA at a higher pressure, the higher emission intensity at lower temperature in this study may be explained by the suppression of vibrational relaxation processes or AIE due to the higher crystal density.^37^ The blue-shifted emission of Et_3_ at higher temperatures also coincides with the shorter emission wavelengths of PA under lower pressures. The intramolecular torsion angles (*θ*_torsion_) and energy frameworks (Fig. S8 and S9, ESI†) correlate the similar spectral shapes of Me_3_ at 123 K and 393 K with a slight change in *E*_InterMol_ (*Δ* = 0.4%) and relatively large *θ*_torsion_ relaxation. In addition to the approximately 5% decrease in *E*_InterMol_ for half-stacked pairs and head-to-head pairs with NH⋯O=C hydrogen bonding in Me_2_Et and Et_3_, respectively, the *E*_InterMol_ between head-to-head pairs without NH⋯O=C interaction in Et_3_ decreased by 25% after heating, which drove the anisotropic expansion of the cell and the appearance of a monomeric emission peak at a higher temperature. Therefore, the intra- and inter-molecular arrangements may change in either ordered or disordered solids depending on the temperature, thus resulting in characteristic emission spectral shifts in accordance with the overlap of the stacking.

These dynamic molecular conformation changes were also confirmed by the temperature-variable FT-IR spectra (Fig. 5c). Amide-I (*ca.* 1630 cm^−1^, C=O stretch), amide-II (*ca.* 1530 cm^−1^, N–H bend/C–N stretch), and amide-A (*ca.* 3000–3500 cm^−1^, N–H stretch) bands were identified,^38–40^ and the shifts in amide-I and II bands (*Δ* ∼ +30 and −40 cm^−1^, respectively) and the appearance of the monomer's amide-A bands (*ca*. 3420 cm^−1^) upon heating above their melting points indicated the breakage of hydrogen bonding, which is in accordance with the spectroscopic and crystallographic results.

## Experimental

### Synthesis

*N-tert*-butyl-2-(alkylsilyl)pyrene-1-carboxamides (alkyl = (CH_3_)_3_: Me_3_, (CH_3_)_2_(C_2_H_5_): Me_2_Et and (C_2_H_5_)_3_: Et_3_) were synthesized following the reported methods.^22^ See ESI† for ^1^H- and ^13^C-NMR, FTIR, and DSC data. ATR-FTIR spectra were performed with a Nicolet 6700 spectrometer equipped with a deuterated triglycine sulphate detector. The spectra were obtained by adding together 64 scans at a resolution of 2 cm^−1^. DSC analyses were performed using a DSC 2500 Discovery, TA Instruments, under a nitrogen atmosphere for all samples at a heating and cooling rate of 10 °C min^−1^.

### Room-temperature MFC spectroscopy

Emission and excitation spectra of pristine powder samples were recorded on a HORIBA Jobin-Yvon Fluorolog FL3-221 spectrometer using a short path length optical quartz cell (20/C/Q/0.2, Starna), and the spectra were corrected for the response of the detector system. The pristine powder (<1 mg) was ground using an agate mortar, and the resulting amorphous solid adhered on a pestle surface was immediately transferred to a quartz plate by smearing to record the spectra within *ca.* 1 min and minimize the effect of self-recovery at room temperature. Due to the small amount of sample and the short time of measurements for the optimized experiments, emission decay lifetimes and quantum yields of the ground forms were not discussed in this study.

### *In situ* temperature-dependent fluorescence spectroscopy

Pristine powders and recovered solids were placed in a THMS600 temperature control stage equipped with a LNP-96 liquid nitrogen pump and a T96-S controller (Linkam Scientific Instruments Ltd). Emission spectra were recorded using an array spectrometer MCPD-9800 and a quantum efficiency measurement system QE-2100 (Otsuka Co., Ltd) in the range between 123 K and 393 K (150 K min^−1^).

### SCXRD experiments

Single crystals were mounted on a glass capillary using Araldite® epoxy adhesive. The measurements were performed on a Rigaku Synergy Custom system with a Hybrid Pixel Array Detector HyPix-Arc 150 using graphite monochromated Mo-Kα radiation. Correction for decay and Lorentz-polarization effects were made using empirical absorption correction, solved by direct methods, and expanded using Fourier techniques. Non-hydrogen atoms were refined anisotropically. Hydrogen atoms were refined using the riding model except for the ones on the amide donor nitrogen atoms which were refined freely. All calculations were performed using the Rigaku CrysAlis(Pro) crystallographic software package. CIF data was confirmed by checkCIF/PLATON service. CCDC data (2285534–2285543: Me_3_, 2285544–2285553: Me_2_Et, 2285523–2285532: Et_3_).†

### PXRD experiments

PXRD patterns were recorded on a Rigaku SmartLab X-ray Diffractometer using a single-crystal Si specimen holder to confirm the crystallinity of the pristine powders (Fig. S4, ESI†).

### Energy framework analysis

The intermolecular interaction energy was calculated at the B3LYP/6-31+G(d,p) level of theory using CrystalExplorer (version 21.5).^27^ The interaction energy was calculated for a target single molecule with all molecules having any atom within 3.8 Å.

### Nanoindentation tests

The target crystals were glued on a glass plate with a Crystalbond™ 509 mounting adhesive using a micromanipulator (Fig. S6, ESI†). Nanoindentations were carried out on a Triboindenter system (Hysitron Inc. Triboindenter TI950) equipped with a 60° 3-sided pyramidal tip on fused quartz (Hysitron Inc. Ti-0038). All experiments were performed in the load-controlled mode using a loading/unloading time of 10 s and a hold time of 10 s at peak load (50–400 μN). More than 4 sets of 81 indentations (9 × 9 array) were performed on each crystal. Young's modulus and hardness were estimated from the unloading curves using the reported eq. 1 and eq. 2 in Fig. S6 (ESI†).^31^*In situ* SPM images were taken using the same probe after indentations.

## Conclusions

In this work, we elucidated the structure–property relationships governing the MFC behaviour using pyrene-derived carboxamide with alkylsilyl and *tert*-butyl groups. All compounds exhibited similar purple-to-blue bathochromic MFC upon grinding. However, the spontaneous fluorescence recovery rates were significantly different, despite their slight differences in the chemical components and molecular packing motifs. Single-crystal XRD experiments and Hirshfeld and energy framework analyses indicated that the chromic direction was determined by the packing motif, whereas the self-recovery process was not simply dominated by the steric hinderance but by the distribution of intermolecular interaction energies. Nanomechanical tests also revealed the reversibility of deformation after unloading, and the “mobility” of molecular solids was demonstrated by temperature-dependent spectroscopy and crystallography either in crystalline or ground/annealed samples. The outcomes of our study contribute to a broader and deeper understanding of the MFC behaviour, including spontaneous fluorescence recovery processes, and inspire further exploration of innovative materials and devices based on simple organic systems.

## Author contributions

YH: conceptualisation, methodology, crystallography, spectroscopy, funding acquisition, writing; AW-P: organic synthesis, characterization; JZ: organic synthesis, characterization; MC: organic synthesis, characterization; TO: nanoindentation experiments; TT: single-crystal and powder XRD experiments; TN: *in situ* temperature-dependent fluorescence spectroscopy; RM: conceptualisation, review and editing; CA: conceptualisation, funding acquisition, review and editing.

## Conflicts of interest

There are no conflicts to declare.

## Supplementary Material

TC-012-D3TC03968D-s001

TC-012-D3TC03968D-s002
