# Correction: Mechanofluorochromism and self-recovery of alkylsilylpyrene-1-carboxamides

**DOI:** 10.1039/d5tc90195b

**Published:** 2025-11-25

**Authors:** Yuichi Hirai, Anna Wrona-Piotrowicz, Janusz Zakrzewski, Magdalena Ciechańska, Takahito Ohmura, Takashi Takeda, Takayuki Nakanishi, Rémi Métivier, Clémence Allain

**Affiliations:** a National Institute for Materials Science (NIMS) 1-1 Namiki Tsukuba 3050044 Japan hirai.yuichi@nims.go.jp; b Department of Organic Chemistry, Faculty of Chemistry, University of Łódź Tamka 12 91-403 Łódź Poland; c Université Paris-Saclay, ENS Paris-Saclay, CNRS, PPSM 4 Avenue des Sciences 91190 Gif-sur-Yvette France remi.metivier@ens-paris-saclay.fr clemence.allain@ens-paris-saclay.fr; d National Institute for Materials Science (NIMS) 1-2-1 Sengen Tsukuba 3050047 Japan

## Abstract

Correction for ‘Mechanofluorochromism and self-recovery of alkylsilylpyrene-1-carboxamides’ by Yuichi Hirai *et al.*, *J. Mater. Chem. C*, 2024, **12**, 1952–1957, https://doi.org/10.1039/D3TC03968D.

The authors regret that in the published article, Fig. 4c contained errors. In addition, in the 3rd paragraph of the Results and discussion section, the sentence: “Thus, the indentation hardness of **Me**_**3**_ was dominated by a more irreversible (plastic) character than the reversible (elastic) ones present in **Me**_**2**_**Et** and **Et**_**3**_” should be replaced with “Thus, the lower indentation resistance of **Me**_**3**_ is attributed to its softer mechanical nature, in comparison with **Me**_**2**_**Et** and **Et**_**3**_”; and in the sentence beginning “In this case…”, the text “possibly resulted in softer and more irreversible characteristics” should be replaced with “possibly resulted in softer characteristics”. The corrected Fig. 4 is as shown in this notice.

**Fig. 1:**
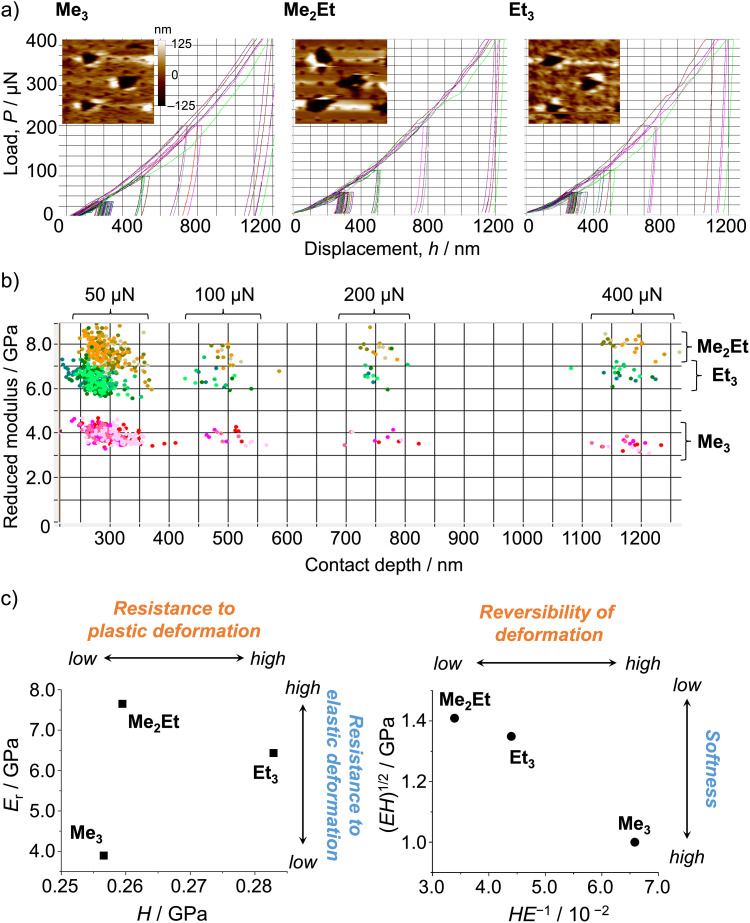
(a) Selected load–displacement curves and *in situ* scanning probe microscopy two-dimensional images after indentations (20 µm × 20 µm). (b) Distribution map of Young's modulus and the contact depth (>300 indentations for each molecule). (c) Young's modulus *versus* the hardness plot (left) and the geometric mean *versus* the elasticity index plot (right).

The Royal Society of Chemistry apologises for these errors and any consequent inconvenience to authors and readers.

