# Supplementary material for: Mechanofluorochromism and self-recovery of alkylsilylpyrene-1-carboxamides
Source: J Mater Chem C Mater. 2024 Jan 19;12(6):1952–7. doi: 10.1039/d3tc03968d (PMC10851921; doi:10.1039/d3tc03968d)
Supplement: TC-012-D3TC03968D-s001 [file TC-012-D3TC03968D-s001.pdf]

Electronic Supplementary Material (ESI) for Journal of Materials Chemistry C.
This journal is © The Royal Society of Chemistry 2023

Supplementary Material (ESI) for Chemical Communications
This journal is © The Royal Society of Chemistry 2001

Supporting Information

Manuscript: "Mechanofluorochromism and Self-Recovery of Alkylsilylpyrene-1-Carboxamides"

Authors: Yuichi Hirai,*[a] Anna Wrona-Piotrowicz,[b] Janusz Zakrzewski,[b] Magdalena Ciechanska,[b] Takahito Ohmura,[d] Takashi Takeda,[a] Takayuki Nakanishi,[a] Rémi Métivier*[c] and Clémence Allain*[c]

This journal is © The Royal Society of Chemistry 2001

Apparatus

^{1}H and ^{13}C NMR spectra were recorded in $CDCl_3$ on a Bruker ARX 600 MHz (600 MHz for ^{1}H and 151 MHz for ^{13}C). Spectra were recorded at room temperature (291 K), chemical shifts are in ppm and coupling constants in Hz.

Me_3: ^{1}H NMR ($CDCl_3$): δ 8.35 (s, 1H), 8.25 (d, J = 9.0, 1H), 8.18 (pseudo-t, J = 6.6, 2H), 8.10 (d, J = 9.6, 1H), 8.08 (d, J = 9.0, 1H), 8.05 (d, J = 9.0, 1H), 8.02 (t, J = 7.8, 1H), 5.74 (s, 1H), 1.64 (s, 9H), 0.55 (s, 9H); ^{13}C NMR ($CDCl_3$): δ 170.56, 138.94, 134.29, 131.45, 131.14, 130.82, 130.47, 128.23, 127.91, 127.44, 127.38, 126.37, 125.44, 125.18, 124.81, 124.38, 124.12, 52.63, 29.06, 0.54.

Me_2Et: ^{1}H NMR ($CDCl_3$): δ 8.33 (s, 1H), 8.25 (d, J = 9.1, 1H), 8.18 (pseudo-t, J = 7.2, 2H), 8.08 (d, J = 9.6, 1H), 8.05 (d, J = 9.0, 1H), 8.02 (d, J = 7.8, 1H), 8.02 (t, J = 7.8, 1H), 5.70 (s, 1H), 1.63 (s, 9H), 1.01–1.06 (m, 5H), 0.53 (s, 6H); ^{13}C NMR ($CDCl_3$): δ 170.56, 139.16, 133.33, 131.51, 131.47, 130.85, 130.42, 128.21, 127.90, 127.49, 127.41, 126.38, 125.44, 125.18, 124.83, 124.41, 124.15, 52.65, 29.06, 8.24, 7.67, –1.83.

Et_3: ^{1}H NMR ($CDCl_3$): δ 8.32 (s, 1H), 8.25 (d, J = 9.0, 1H), 8.18 (t, J = 6.6, 2H), 8.10 (d, J = 9.6, 2H), 8.05 (d, J = 9.0, 1H), 8.01 (d, J = 7.2, 1H), 5.66 (s, 1H), 1.64 (s, 9H), 1.14–1.10 (m, 6H), 1.05–1.02 (m, 9H); ^{13}C NMR ($CDCl_3$): δ 170.57, 139.48, 132.22, 131.46, 131.29, 130.84, 130.28, 128.13, 127.85, 127.55, 127.45, 126.36, 125.78, 125.43, 125.16, 124.81, 124.40, 124.19, 52.66, 28.96, 7.70, 4.05.

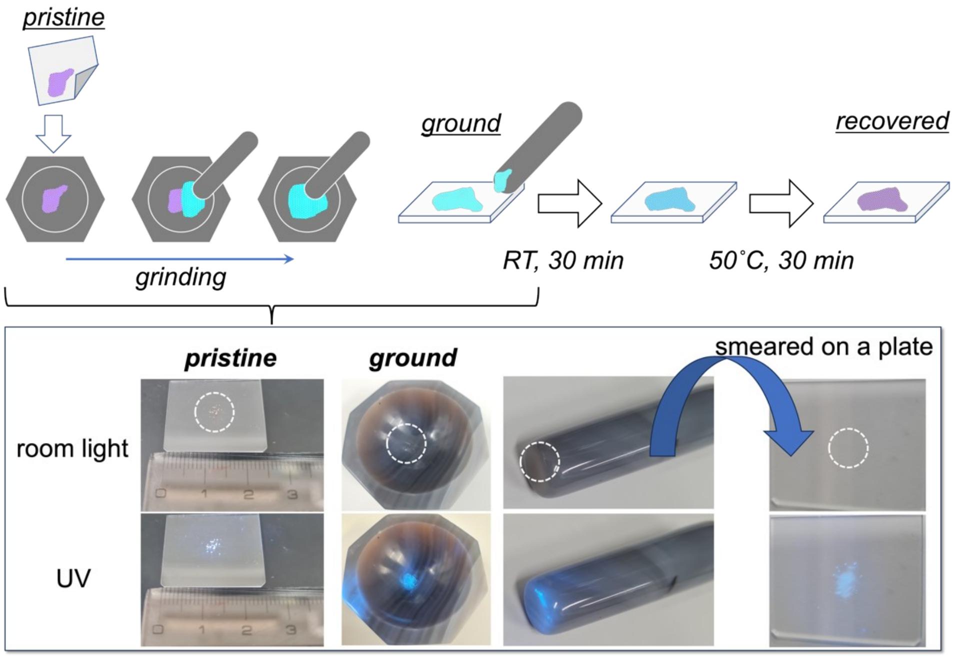

This journal is © The Royal Society of Chemistry 2001

Figure S1. A sample preparation procedure for MFC spectroscopy (at 23˚C, 43%RH). White dotted circles show the sample positions under a room light.

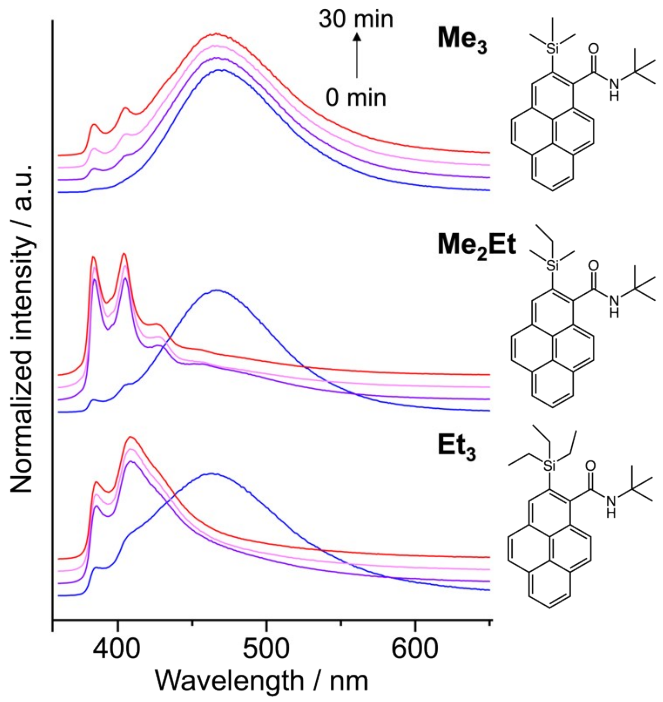

Figure S2. Normalised emission spectra of $\mathbf{Me_3}$, $\mathbf{Me_2Et}$, and $\mathbf{Et_3}$ at room temperature (RT) after grinding (blue: 0 min (ground), purple, pink, and red: 10, 20, and 30 min at RT after grinding, respectively, λ_{ex} = 340 nm, in the solid state).

This journal is © The Royal Society of Chemistry 2001

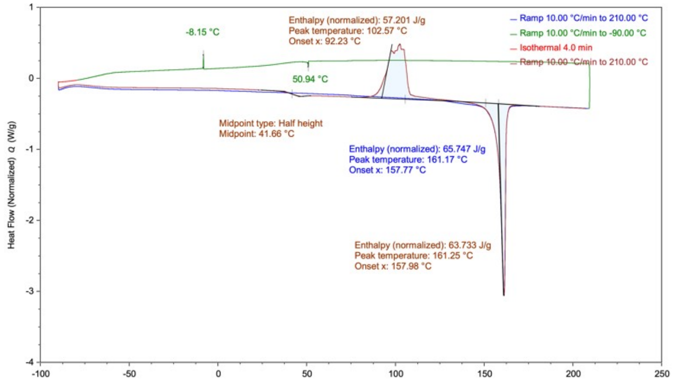

Me$_3$

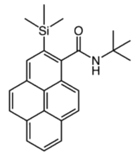

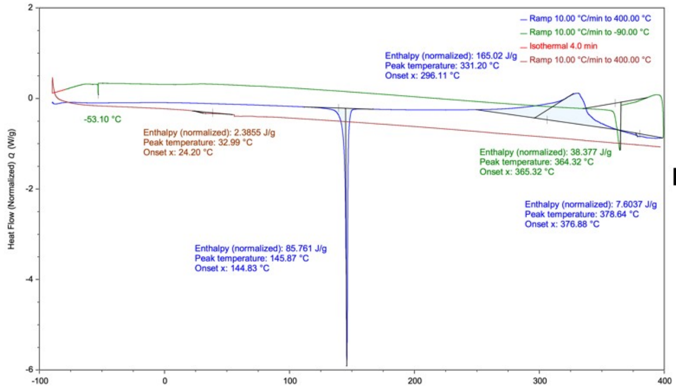

Me$_2$Et

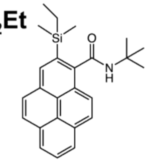

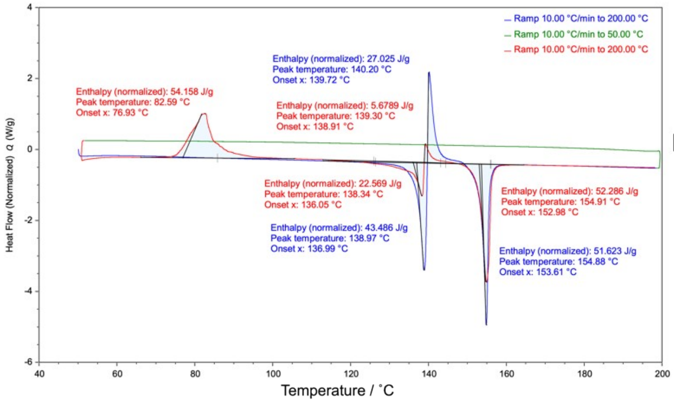

Et$_3$

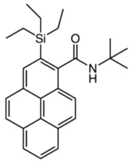

This journal is © The Royal Society of Chemistry 2001

Figure S3. DSC thermograms of **Me_3**, **Me_2Et**, and **Et_3**.

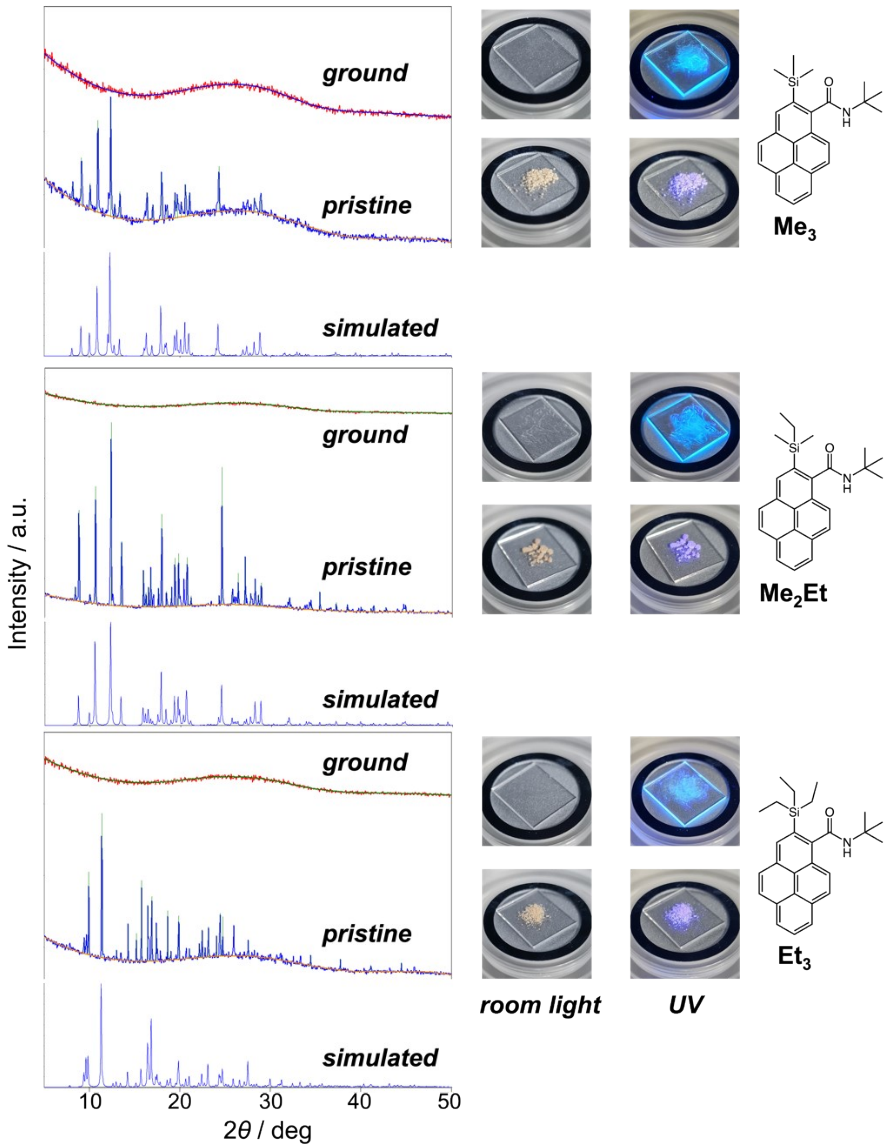

Supplementary Material (ESI) for Chemical Communications
This journal is © The Royal Society of Chemistry 2001

Figure S4. Simulated and experimental (pristine and ground) PXRD patterns and photos under room light and UV irradiation of $\mathbf{Me_3}$, $\mathbf{Me_2Et}$, and $\mathbf{Et_3}$.

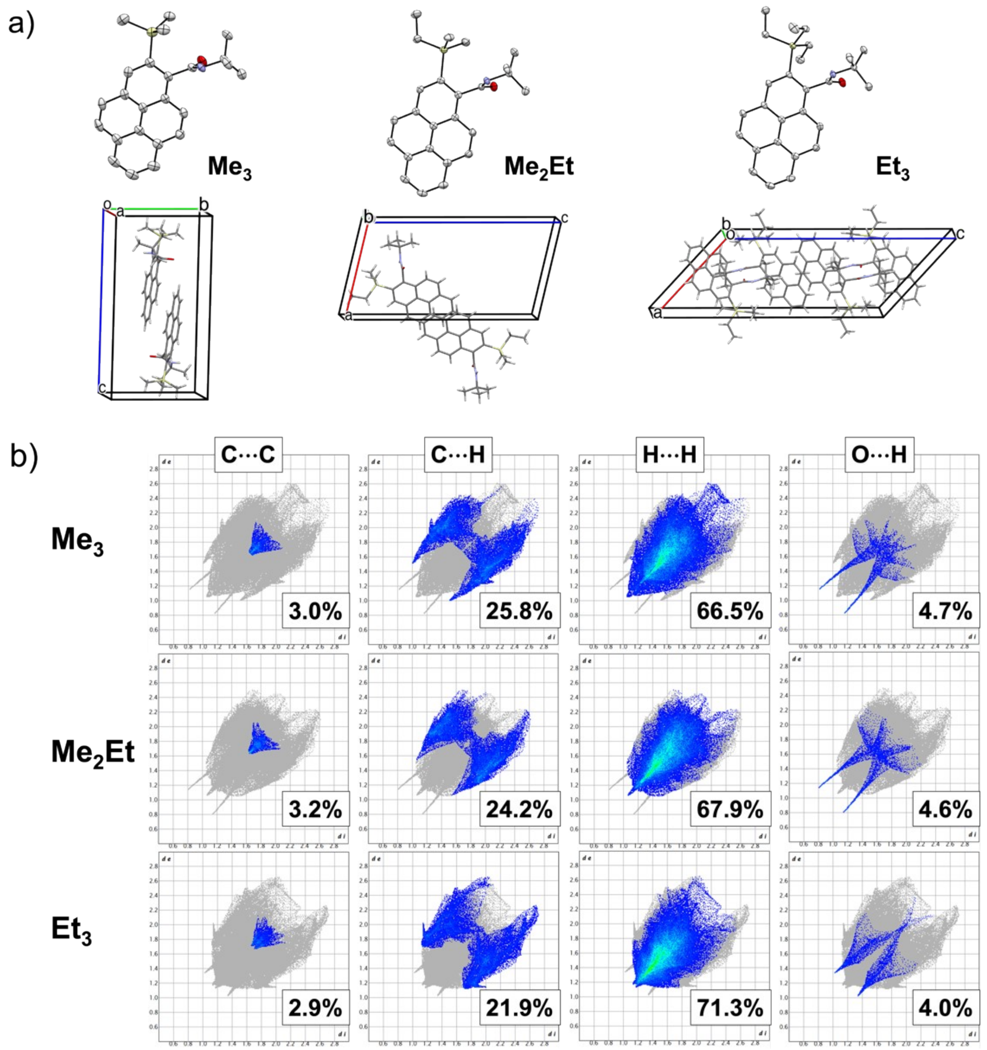

Figure S5. a) ORTEP view (top, thermal ellipsoids are shown at 50% probability and hydrogen atoms are omitted for clarity) and packing diagram of two neighboring molecules (bottom) and b) Hirshfeld surfaces with fingerprint plots for specific pairs of atom-

types of **Me_3**, **Me_2Et**, and **Et_3** (grey shadow: outline of the complete fingerprint plot, d_i and d_e: distances from the Hirshfeld surfaces to the nearest nucleus inside and outside the surface, respectively).

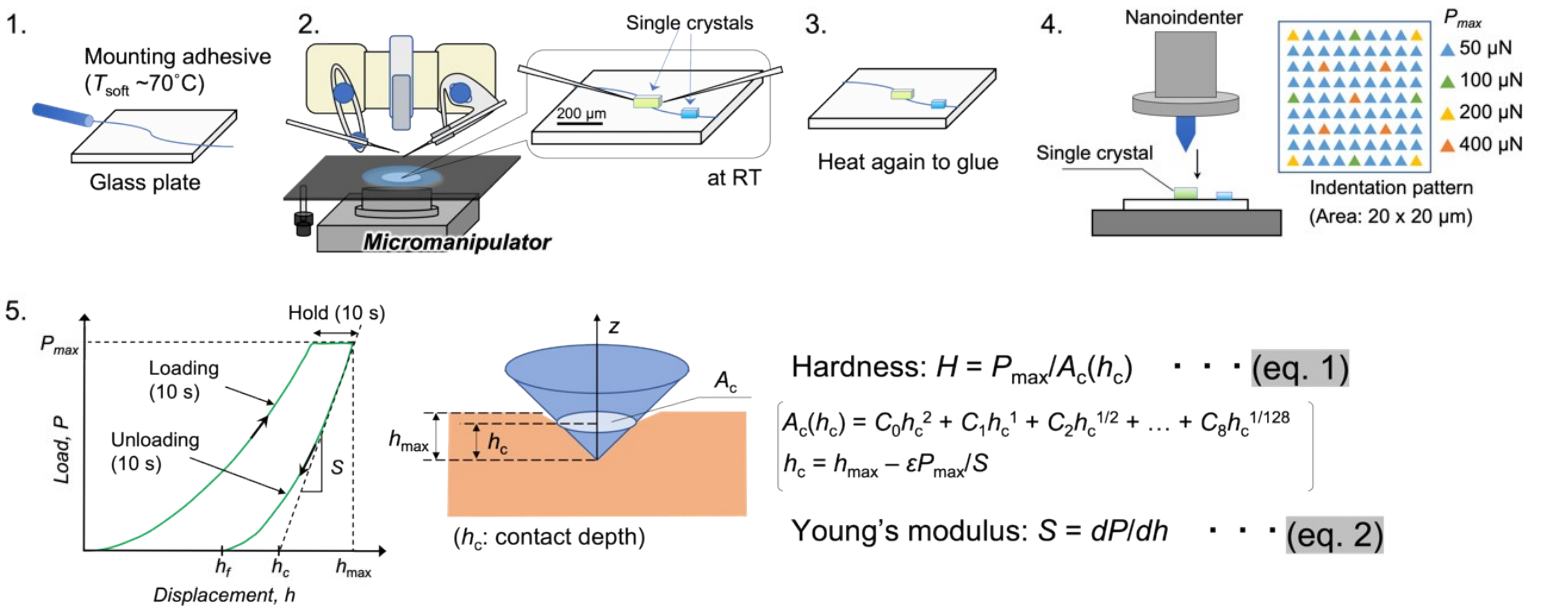

Figure S6. Schematic of nanomechanical tests.

This journal is © The Royal Society of Chemistry 2001

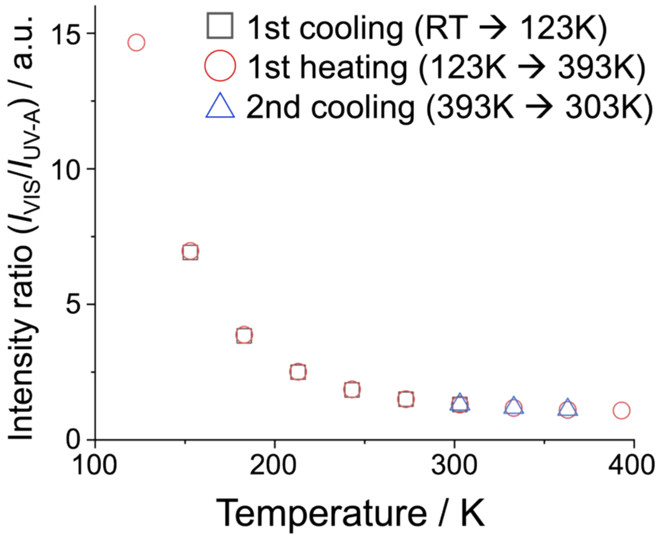

Figure S7. Emission intensity ratios between VIS (λ_{em} ~ 410–419 nm) and UV (λ_{em} ~ 388 nm) bands in **Et_3** upon cooling and heating processes (plots were overlapped).

This journal is © The Royal Society of Chemistry 2001

Table S1. Summary of selected crystallographic parameters of **Me_3** in temperature-variable SCXRD experiments.

T / K	123 K	153 K	183 K	213 K	243 K	273 K	303 K	333 K	363 K	393 K
$D_{calc.}$/ g cm^{-3}	1.197	1.185	1.181	1.169	1.162	1.153	1.146	1.134	1.126	1.119
μ/mm^{-1}	0.126	0.125	0.125	0.123	0.123	0.122	0.121	0.120	0.119	0.118
a/Å	10.9996	11.0400	11.0479	11.0892	11.1134	11.1497	11.1876	11.2381	11.2812	11.3306
b/Å	9.9013	9.8882	9.8798	9.8918	9.8968	9.9080	9.9160	9.9380	9.9489	9.9451
c/Å	19.2782	19.4597	19.5539	19.6740	19.7565	19.8583	19.9362	20.0348	20.1153	20.1886
α/°	90	90	90	90	90	90	90	90	90	90
β/°	99.065	99.626	100.010	100.410	100.743	101.193	101.696	102.077	102.514	102.876
γ/°	90	90	90	90	90	90	90	90	90	90
V/Å^3	2073.38	2094.43	2101.84	2122.55	2134.87	2152.03	2165.72	2188.04	2204.01	2217.69
Z	4	4	4	4	4	4	4	4	4	4
Z’	1	1	1	1	1	1	1	1	1	1
Q_{min}/°	2.140	2.317	2.317	2.312	2.098	2.306	2.304	2.298	2.295	2.295
Q_{max}/°	26.372	26.363	26.371	26.370	26.369	26.372	26.367	26.373	26.372	26.371
Measured Refl.	49858	50991	48366	54038	51892	48310	51373	48414	48512	50075
Independent Refl.	4248	4292	4312	4349	4373	4404	4433	4473	4502	4528
Refl. with I > 2(I)	3755	3525	3353	3182	3186	2944	3079	2653	2782	2560
R_{int}	0.0401	0.0338	0.0383	0.0308	0.0269	0.0382	0.0263	0.0397	0.0502	0.0597
GooF	1.067	1.064	1.061	1.065	1.072	1.086	1.688	1.074	1.077	1.235
wR_2 (all data)	0.1157	0.1479	0.1769	0.1952	0.2306	0.2519	0.2374	0.2429	0.2857	0.3475
wR_2	0.1121	0.1391	0.1651	0.1786	0.2063	0.2219	0.2224	0.2148	0.2503	0.3010

R_1 (all data)	0.0429	0.0600	0.0720	0.0791	0.0862	0.1000	0.0817	0.0943	0.0981	0.1162
R_1	0.0386	0.0505	0.0592	0.0620	0.0660	0.0725	0.0619	0.0589	0.0698	0.0851

*Formula: $C_{24}H_{27}NOSi$, Formula weight: 373.55, Color: clear colorless, Shape: needle, Size: 0.81 × 0.11× 0.08 mm^3, Crystal system: monoclinic, Space group: $P2_1/c$, Wavelength: 0.71073 Å, Radiation type: Mo Kα for all measurements

Table S2. Summary of selected crystallographic parameters of **Me_2Et** in temperature-variable SCXRD experiments.

T / K	123 K	153 K	183 K	213 K	243 K	273 K	303 K	333 K	363 K	393 K
$D_{calc.}$/ g cm^{-3}	1.199	1.194	1.189	1.184	1.178	1.171	1.166	1.156	1.147	1.138
μ/mm^{-1}	0.124	0.124	0.123	0.123	0.122	0.121	0.121	0.120	0.119	0.118
a/Å	10.7771	10.8011	10.8255	10.8510	10.8797	10.9116	10.9394	10.9844	11.0196	11.0625
b/Å	9.85420	9.86550	9.8799	9.8897	9.9095	9.9285	9.9405	9.9710	9.9958	10.0230
c/Å	20.7765	20.7927	20.8127	20.8306	20.8527	20.8812	20.8935	20.9290	20.9717	21.0259
α/°	90	90	90	90	90	90	90	90	90	90
β/°	103.243	103.322	103.394	103.454	103.511	103.567	103.629	103.709	103.749	103.939
γ/°	90	90	90	90	90	90	90	90	90	90
V/Å^3	2147.78	2156.02	2165.48	2174.06	2185.97	2199.07	2208.06	2226.97	2243.82	2262.69
Z	4	4	4	4	4	4	4	4	4	4
Z'	1	1	1	1	1	1	1	1	1	1
Q_{min}/°	2.299	2.297	2.294	2.292	2.009	2.284	2.281	2.003	2.270	2.264
Q_{max}/°	26.371	26.370	26.370	26.371	26.372	26.372	26.371	26.371	26.372	26.371
Measured Refl.	52547	55187	50407	53370	55529	55631	53774	56494	54290	59552
Independent Refl.	4392	4408	4433	4449	4472	4506	4525	4568	4603	4638
Refl. with I > 2(I)	4015	4022	3973	3946	3903	3904	3828	3791	3675	3549

R_{int}	0.0326	0.0333	0.0303	0.0311	0.0282	0.0270	0.0362	0.0272	0.0310	0.0252
GooF	1.122	1.060	1.065	1.074	1.064	1.077	1.081	1.105	1.080	1.091
wR_2 (all data)	0.1200	0.1126	0.1164	0.1208	0.1254	0.1354	0.1504	0.1632	0.1944	0.2206
wR_2	0.1126	0.1099	0.1124	0.1168	0.1204	0.1305	0.1435	0.1546	0.1801	0.2043
R_1 (all data)	0.0455	0.0411	0.0430	0.0444	0.0455	0.0488	0.0536	0.0571	0.0688	0.0741
R_1	0.0407	0.0384	0.0390	0.0402	0.0409	0.0439	0.0471	0.0495	0.0549	0.0633

*Formula: $C_{25}H_{29}NOSi$, Formula weight: 387.58, Color: clear colorless, Shape: block, Size: 0.64 × 0.22× 0.17 mm^3, Crystal system: monoclinic, Space group: $P2_1/c$, Wavelength: 0.71073 Å, Radiation type: Mo Kα for all measurements

Table S3. Summary of selected crystallographic parameters of **Et$_3$** in temperature-variable SCXRD experiments.

T / K	123 K	153 K	183 K	213 K	243 K	273 K	303 K	333 K	363 K	393 K
$D_{calc.}$/ g cm^{-3}	1.193	1.186	1.179	1.173	1.166	1.159	1.150	1.144	1.135	1.124
μ/mm^{-1}	0.120	0.119	0.118	0.118	0.117	0.116	0.116	0.115	0.114	0.113
a/Å	11.8370	11.8802	11.9292	11.9667	12.0081	12.0430	12.0890	12.1154	12.1424	12.1689
b/Å	10.4643	10.4692	10.4691	10.4761	10.4878	10.5024	10.5330	10.5447	10.5631	10.5841
c/Å	25.3373	25.3536	25.3570	25.3546	25.3608	25.3746	25.3765	25.3643	25.3408	25.2785
α/°	90	90	90	90	90	90	90	90	90	90
β/°	132.506	132.411	132.332	132.251	132.157	132.090	132.040	131.856	131.547	131.031
γ/°	90	90	90	90	90	90	90	90	90	90
V/Å^3	2313.70	2328.3	2341.1	2352.8	2367.7	2381.7	2399.8	2413.5	2432.5	2456.1
Z	4	4	4	4	4	4	4	4	4	4
Z'	1	1	1	1	1	1	1	1	1	1
Q_{min}/°	2.181	2.176	2.228	2.170	2.224	2.220	2.161	2.212	2.207	2.136

Q_{max}/°	26.371	26.370	26.372	26.371	26.370	26.371	26.370	26.370	26.371	26.372
Measured Refl.	53032	56456	56871	58560	54155	57295	57587	51420	54877	56273
Independent Refl.	4722	4755	4778	4804	4834	4870	4901	4933	4977	5023
Refl. with I > 2(I)	4372	4366	4331	4311	4259	4240	4196	4097	3876	3568
R_{int}	0.0315	0.0320	0.0324	0.0320	0.0335	0.0303	0.0285	0.0609	0.0272	0.0265
GooF	1.081	1.085	1.073	1.084	1.069	1.089	1.102	1.142	1.076	1.217
wR_2 (all data)	0.1147	0.1155	0.1188	0.1257	0.1291	0.1328	0.1476	0.1837	0.2092	0.3004
wR_2	0.1126	0.1132	0.1160	0.1222	0.1249	0.1291	0.1426	0.1773	0.1968	0.2765
R_1 (all data)	0.0415	0.0418	0.0424	0.0452	0.0464	0.0486	0.0540	0.0596	0.0731	0.0999
R_1	0.0390	0.0393	0.0394	0.0417	0.0420	0.0439	0.0478	0.0539	0.0628	0.0853

*Formula: $C_{27}H_{33}NOSi$, Formula weight: 415.63, Color: clear light brown, Shape: block, Size: 0.51 × 0.41× 0.26 mm^3, Crystal system: monoclinic, Space group: $P2_1/c$, Wavelength: 0.71073 Å, Radiation type: Mo Kα for all measurements

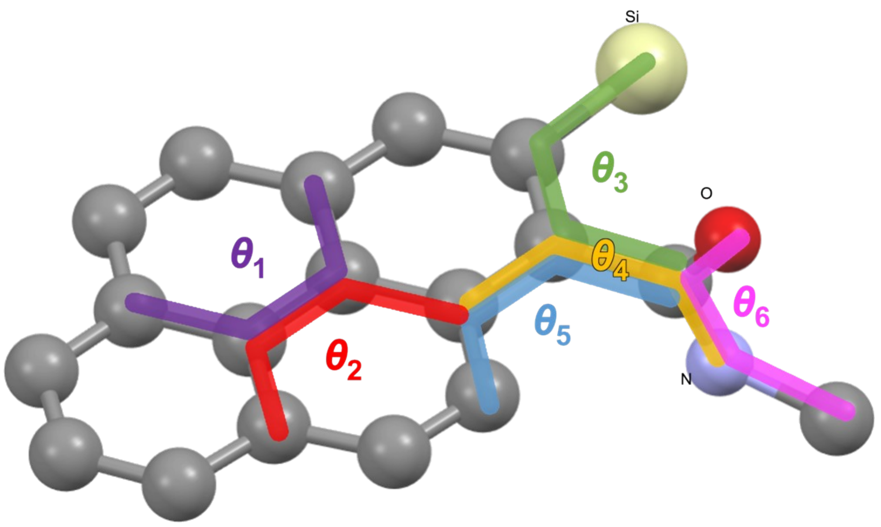

		θ_1 / °	θ_2 / °	θ_3 / °	θ_4 / °	θ_5 / °	θ_6 / °
**Me_3**	123 K	0.97	1.01	2.91	79.4	1.14	0.03
	393 K	0.24	2.04	0.87	87.4	1.28	0.60
**Me_2Et**	123 K	0.24	1.49	1.15	87.2	1.31	0.67
	393 K	0.01	2.08	1.37	88.3	0.89	1.86
**Et_3**	123 K	0.94	1.58	12.8	81.5	4.08	4.12
	393 K	0.07	2.67	9.80	87.8	4.36	3.50

Figure S8. Selected intramolecular torsion angles of **Me_3**, **Me_2Et**, and **Et_3** at 123 K and 393 K (methyl and ethyl groups and hydrogen atoms were omitted for clarity).

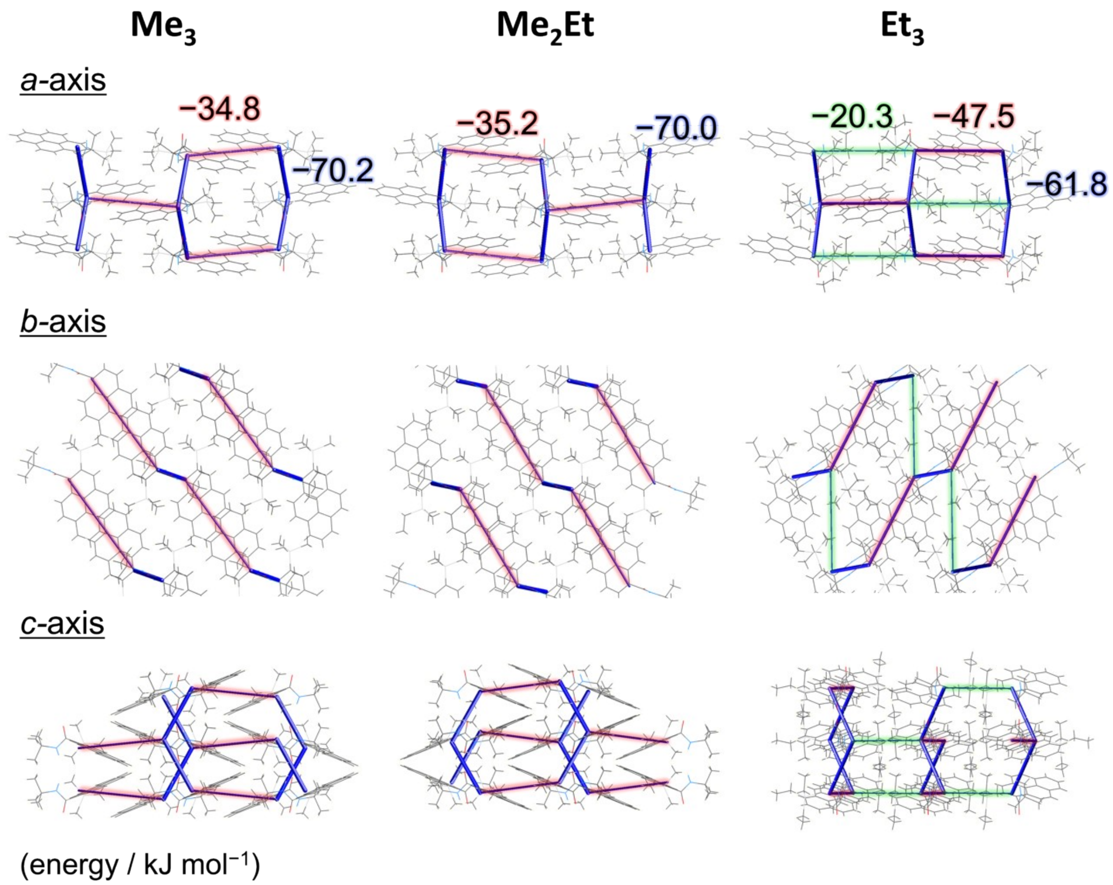

Figure S9. CE-B3LYP energy frameworks of **Me$_3$**, **Me$_2$Et**, and **Et$_3$** from different perspectives representing the net interaction energy (cut-off: 20.0 kJ mol^{-1}) at 393 K. The blue cylinders are connecting ceters of mass of adjacent molecules, and the diameter of cylinders are proportional to the magnitutde of energy. Opaque red and green shadows highlight the interactions between parallel half-stacked pairs and non-parallel head-to-head pairs without NH···O=C hydrogen bonding.
